# MRI characteristics of appendicular soft tissue lymphoma: a retrospective analysis

**DOI:** 10.1186/s12957-026-04478-1

**Published:** 2026-07-09

**Authors:** Valentin Weisse, Sebastian Weiss, Peter Bannas, Alexander Korthaus, Alonja Reiter, Thore Raschka, Andreas Lübke, Carsten Schlickewei, Alexander Spiro, Jana Käthe Striefler, Karl-Heinz Frosch, Matthias Priemel

**Affiliations:** 1 Department of Trauma and Orthopaedic Surgery, University Medical Center Hamburg- Eppendorf, Hamburg, Germany; 2 Department of Orthopaedic Surgery, Trauma Surgery, and Sports Medicine, Cologne Merheim Medical Center, Witten/Herdecke University, Ostmerheimer Str. 200, Cologne, 51109 Germany; 3 Department of Diagnostic and Interventional Radiology and Nuclear Medicine, University Medical Center Hamburg-Eppendorf, Hamburg, Germany; 4 Institute of Pathology, University Medical Center Hamburg-Eppendorf, Hamburg, Germany; 5 Department of Trauma Surgery, Orthopaedics and Sports Traumatology, BG Klinikum Hamburg, Hamburg, Germany; 6 Department of Pediatric Orthopaedic Surgery, Children’s Hospital Hamburg-Altona, Hamburg, Germany; 7 Department of Internal Medicine II, Oncology/Hematology/BMT/Pneumology, University Medical Center Hamburg-Eppendorf, Hamburg, Germany

**Keywords:** Soft tissue lymphoma, MRI, Soft tissue masses, Extremities, MSK

## Abstract

**Objectives:**

Appendicular soft tissue lymphoma (ASTL) are rare and frequently misinterpreted as soft tissue sarcoma. This can put patients at risk. Existing studies evaluating MRI of ASTL are sparse, small scaled and report heterogenous results. The purpose of this study was therefore, to identify comprehensive MRI characteristics of ASTL.

**Material & methods:**

We retrospectively analyzed demographic data, clinical presentation and MRI data of 26 patients presenting to our musculoskeletal tumor surgery center between 2011 and 2023 with histopathologically proven ASTL.

**Results:**

Identified MRI characteristics included a diameter larger than 5 cm (77%), hyperintense signal intensity in all T2-weighted, PD-weighted and short tau inversion recovery (STIR) sequences and iso- to slightly hyperintense signal intensity in T1-weighted sequences compared to skeletal muscle. Furthermore, irregular contrast enhancement of adjacent fasciae (60%) and predominant enhancement of peripheral tumor accompanied by slightly less intensive homogenous enhancement of the remaining tumor (55%) were observed. Additionally, regional lymphadenopathy (31%), subcutaneous stranding (58%), traversing vessels (88%) and the absence of encapsulation (100%) were discernable. Features seen in ASTL primarily involving muscle (*n* = 16), were involvement of multiple muscles (100%), tissue types (88%) and anatomical compartments (69%) as well as long segmental involvement (69%), growth along neurovascular bundles (56%) and partial or complete encasement of major vessels (81%). Intratumoral necrosis was identified in four cases (25%).

**Conclusion:**

MRI provides the presented morphological indicators, that should raise suspicion of ASTL. Our findings suggest that necrosis may occasionally occur in pretreatment ASTL and its presence alone should not exclude lymphoma from the differential diagnosis.

## Key message

1. Appendicular soft tissue lymphoma are rare and frequently misinterpreted as soft tissue sarcoma, which can put patients at risk of inadequate therapy.

2. MRI provides morphological indicators for the presence of appendicular soft tissue lymphoma.

3. The presence of necrosis alone should not exclude soft tissue lymphoma from the differential diagnosis in soft tissue tumor presentations.

## Introduction

Primary lymphoma of the appendicular soft tissues (ASTL) represent a rare subentity, which account for merely 0.1% of lymphoma patients [1]. Moreover, only 1.2 to 2% of all soft tissue tumors are soft tissue lymphoma [2, 3]. Soft tissue sarcoma (STS) is more prevalent (20% of all soft tissue tumors) than soft tissue lymphoma and represents the most frequently suspected diagnosis in soft tissue lymphoma presentations [4–6]. Lymphoma themselves are oftentimes not considered in the differential diagnosis of soft tissue masses [7]. ASTL usually responds to the application of a chemotherapy regime, whereas STS is usually managed by performing radical excisional surgery [8–10]. Performing major surgery in ASTL management would be highly inappropriate and would put patients at risk for deficient function and complications. Given the deviant treatment options and associated risks, the correct differential diagnosis is of utmost importance for adequate therapy.

MRI is the non-invasive imaging modality of choice to evaluate and distinguish between soft tissue masses [11–14] and to assist the pathologist in adjusting the implemented immunohistological stains [15].

Nevertheless, a recently conducted systematic review identified a mere total of 77 described cases in which MRI characteristics of ASTL were investigated [16]. Moreover, it was shown that existing studies are scarce, mostly small scaled or case reports, report heterogenous results and often lack structure and quality [16, 17].

Therefore, the purpose of this study was to identify comprehensive MRI characteristics suggestive of ASTL presence in the largest single-center study population to date.

## Materials and methods

This retrospective analysis was approved by the local ethics committee (No. of registration: WF-085/21). Informed consent of patients was waived due to the retrospective and fully anonymized design of the presented study. Moreover, the presented study was conducted and reported in compliance with the Strengthening the Reporting of Observational Studies in Epidemiology (STROBE) guidelines [18].

### MRI protocols

Due to the tertiary referral status of our unit, MRI was performed using a variety of systems. Field strengths were either 1.5 (*n* = 20) or 3 (*n* = 6) Tesla.

Protocols included T1-weighted turbo spin echo (T1w) images (*n* = 24), T2-weighted turbo spin echo (T2w) images (*n* = 21), fat suppressed T1w images without application of contrast agent (*n* = 5) and with application of contrast agent (*n* = 16). In four cases, non-fat-suppressed T1w contrast enhanced images were obtained. Short tau inversion recovery sequences (STIR) were acquired in seven cases and proton density weighted (PDw) images were attained in 14 cases.

### MRI analysis

To ensure interrater reliability, all analyses were conducted by two independent investigators.

Differences in the resulting data sheets were resolved through a tie-breaking radiologist co-author. Analyzed MRI features included lesion size, signal intensity in T1w, T2w-, PDw, fat-suppressed T1w and STIR images compared to adjacent muscle. Furthermore, we evaluated contrast enhanced behavior of tumor and adjacent fascial planes, demarcation patterns, number of involved muscles, involved anatomical compartments and involved tissue types. As classical anatomical compartments are typically not defined in the shoulder and back regions, the deltoid muscle, the non-autochthonous and the autochthonous back muscles were considered as one compartment each. In the thigh/iliac region, the iliopsoas muscle was considered as a single compartment.

Moreover, we analyzed the presence of long segmental involvement (growth orientated along muscle fascicles), subcutaneous stranding (reticular signal alteration of subcutaneous tissue), growth along or encasement of neurovascular bundles, osseous involvement, the presence of traversing vessels, encapsulation and intratumoral necrosis. Necrosis was thereby defined as a lesion showing low to intermediate signal intensity compared to surrounding tissue in T1w sequences, high signal intensity in water sensitive sequences and the absence of contrast agent enhancement.

### Patients and study design

Patient records and MR imaging of patients who were referred to our tertiary center for musculoskeletal tumor surgery from 2011 to 2023 were retrospectively reviewed for patients presenting with histopathologically proven ASTL manifestation. Clinical and demographic data were extracted from patient records. These data included the radiologically suspected diagnosis, age, sex, histological diagnosis, localization (determined by the localization containing the largest extent of tumor if several regions were affected), the presence of regional lymphadenopathy, B symptoms and elevation of lactate dehydrogenase levels (LDH). LDH levels were considered elevated at > 245 U/L [19].

Due to suspected atypical imaging features, we excluded patients with recurrence of disease and immunocompromised patients with conditions such as acquired immunodeficiency syndrome (AIDS) [20–24]. To avoid inclusion of primary osseous lymphoma with spread to the soft tissues, exclusively mild signal intensity abnormalities of bone marrow accompanied by significantly more extensive soft tissue involvement were included [25]. The conducted search resulted in 59 patients presenting with lymphoma manifestation, of whom 25 were excluded due to primary osseous manifestation. Furthermore, four patients were excluded due to recurrent disease and in four patients MR imaging was not available. A total of 26 patients was included.

### Statistical analysis

Acquired data were evaluated by using descriptive statistics (absolute and relative frequencies).

When adequate, a standard deviation was calculated. Given the variety of used protocols, the number of patients included in the statistical analysis of parameters shows fluctuation. In case of incomplete information, statistics were calculated excluding the affected individual from the calculation of the respective variable. Furthermore, a subgroup analysis comparing primary intramuscular appendicular lymphoma (PML) and primary subcutaneous appendicular lymphoma (PSCL) was performed, wherein allocation was determined measuring the largest extent of tumor in each tissue. In instance of missing statistical significance, the subgroups were conjoined for final analysis. A Fishers’ exact test was utilized to calculate significance of binary data and a two tailed Mann-Whitney U test was deployed to assess numerical polytomous data. A value of *p* < 0.05 was considered statistically significant. Statistically non-significant but still notable differences between the subgroups were disclosed if they exceeded 20%. Moreover, we reported effect sizes as absolute differences between PML and PSCL with corresponding 95% confidence intervals (CI). These were calculated using the Newcombe/Wilson score method. Statistical analyses were performed using GraphPad Prism V.9 (San Diego, CA, USA).

## Results

Our study included a total of 26 patients with histopathologically proven ASTL. Mean age was 68 ± 15 years (range 17–85). 16 patients were female (62%).

STS was the most frequently suspected diagnosis by the initially assessing radiologist. It was among the articulated differential diagnoses in 16 of 23 cases (70%). Histologically, diffuse large B-cell lymphoma represented the most frequently identified subentity (*n* = 15; 58%). Demographic, clinical and diagnostical data are displayed in Table 1.

**Table 1:** Demographic and clinical data of included patients

Patient no.	Demographic Data		Clinical Data				
	Age (years)	Sex	Radiologically Suspected Differential Diagnosis	Histological Diagnosis	Localization	Regional Lymphadenopathy	Pain or Discomfort
1	85	W	Sarcoma	Diffuse large B-cell lymphoma	Shoulder	Yes	Yes
2	81	M	Histiocytoma, Metastasis	Primary cutaneous large B-Cell lymphoma	Thigh	No	No
3	79	W	Sarcoma	Diffuse large B-cell lymphoma	Thigh	Yes	No
4	71	W	Hematoma	Follicular lymphoma	Thigh	No	No
5	57	W	Sarcoma	Diffuse large B-cell lymphoma	Back	No	Yes
6	68	W	N/A	Diffuse large B-cell lymphoma	Thigh	Yes	No
7	73	M	Sarcoma	Marginal zone lymphoma	Upper Arm	No	No
8	66	M	Malignant Process	Follicular lymphoma	Psoas Muscle	No	Yes
9	74	W	Sarcoma, Lymphoma	Diffuse large B-cell lymphoma	Shoulder	No	No
10	64	M	Sarcoma	Diffuse large B-cell lymphoma	Psoas Muscle	No	Yes
11	85	W	Sarcoma	Diffuse large B-cell lymphoma	Thigh	Yes	Yes
12	17	W	N/A	ALK positive anaplastic large cell lymphoma	Thigh	No	Yes
13	75	M	Sarcoma	Follicular lymphoma	Upper Arm	Yes	No
14	73	W	Sarcoma, Desmoid tumor	Follicular lymphoma	Thigh	No	Yes
15	72	M	Sarcoma	Diffuse large B-cell lymphoma	Thigh	Yes	No
16	83	M	N/A	Diffuse large B-cell lymphoma	Thigh	No	Yes
17*	70	W	Sarcoma, Schwannoma, Atheroma	Diffuse large B-cell lymphoma	Thigh*	No	No
18*	66	M	Lymphoma, Sarcoma	Follicular lymphoma	Thigh*	No	No
19*	44	W	Schwannoma	Diffuse large B-cell lymphoma	Upper Arm*	Yes	Yes
20*	85	W	Sarcoma	Diffuse large B-cell lymphoma	Calf*	No	No
21*	47	M	Lymphoma	Diffuse large B-cell lymphoma	Shoulder*	No	No
22*	76	W	Sarcoma	Follicular lymphoma	Thigh*	No	No
23*	67	M	Sarcoma, Lymphoma	Marginal zone lymphoma	Pectoral Muscles*	Yes	Yes
24*	57	W	Schwannoma	Diffuse large B-cell lymphoma	Forearm*	No	No
25*	55	W	Fibroma	Diffuse large B-cell lymphoma	Upper Arm*	No	Yes
26*	75	W	Sarcoma	Marginal zone lymphoma	Thigh	No	Yes
Total	Mean ± SD: 68 ± 15 Range: 17–85	16 W(62%) 10 M (38%)	11 Sarcoma 3 Sarcoma, Lymphoma 2 Schwannoma 1 Lymphoma 1 Sarcoma, Schwannoma, Atheroma 1 Sarcoma, Desmoid tumor 1 Fibroma 1 Hematoma 1 Malignant Process 1 Histiocytoma, Metastasis 3 N/A ^#^	15 Diffuse large B-cell lymphoma 6 Follicular lymphoma 3 Marginal zone lymphoma 1 Anaplastic large T-cell lymphoma 1 Cutaneous large B- cell lymphoma	13 Thigh 4 Upper Arm 3 Shoulder 2 Psoas Muscle 1 Calve 1 Back 1 Pectoral Muscles 1 Forearm	8 Yes (31%) 18 No (69%)	12 Yes (46%) 14 No (54%)

*subcutaneous manifestations

^#^ Not Applicable

Tumor manifestation was mainly located in the proximal extremities (thigh and psoas muscle: *n* = 15; 58%, upper arm and shoulder: *n* = 7; 27%). Regional lymphadenopathy was identified in eight cases (31%) and pain or discomfort were reported in twelve cases (46%). B symptoms were reported in two cases (8%). LDH levels were assessed in 18 cases and showed statistically significant differences between the subgroups (Table 2), as elevated levels were found in nine of 11 PML (82%), whereas one of seven PSCL (14%) showed increased LDH levels (*p* = 0.012 ; CI = 20.2 to 85.1). Results of acquired MRI data are displayed in Table 3. The mean largest lesion diameter was 10.7 ± 7.1 cm. The largest lesion measured 35.0 cm, whereas the smallest lesion measured 1.0 cm. 20 (77%) lesions surpassed a 5 cm diameter (T2 in UICC TNM classification).

**Table 2:** Statistically significant differences between primary intramuscular appendicular lymphoma and primary subcutaneous appendicular lymphoma

Feature	Primary Subcutaneous Lymphoma (PSCL; *n* = 10)	Intramuscular Lymphoma (PML; *n* = 16)	*P*-Value^#^	Absolute difference PML − PSCL, %	95% CI
Elevated LDH Levels	1/7 (14%) x̅=213 u/L Range: 152–464 u/L	9/11 (82%) x̅=448 u/L Range: 201–805 u/L	0.012	68%	20.2 to 85.1
Multiple Involved Muscles	1/4*(25%) x̅=1.25 Range: 1–2	16/16 (100%) x̅=4.9 Range: 1–10	0.003	75%	26.1 to 95.4
Multiple Involved Compartments	0/4* x̅=1,0 Range: 1–1	11/16 (69%) x̅=2.0 Range:1–4	0.026	69%	14.0 to 85.8
Multiple Tissue Types	4/10 (40%) x̅=1,5 Range 1–3	14/16 (88%) x̅=2,0w Range 1–4	0.025	48%	10.4 to 72.4
Long Segmental Involvement	0/4*	11/16 (69%)	0.026	69%	14.0 to 85.8
Growth along Neurovascular Bundles	1/10 (10%)	9/16 (56%)	0.036	46%	8.1 to 68.5
Encasement of Major Vessels	4/10 (40%)	13/16 (81%)	0.046	41%	3.6 to 67.4

* Primary subcutaneous ASTL infiltrating adjacent skeletal muscle

^#^ Values < 0.05 were classified as statistically significant

x̅: Mean value

**Table 3:** MRI characterization of lymphoma by signal intensity, contrast patterns, and structural involvement

Patient no.	T1w^1^	T2w^1^	PDw^1^	STIR^1^	Pattern of Contrast Enhancement	No. of Involved Compartments	Growth along Neurovascular Bundles	Subcutaneous Stranding
1	Isointense	Hyperintense	Hyperintense	N/A	Homogeneous	2	Yes	No
2	Isointense	N/A	Hyperintense	N/A	Peripheral & Homogenous	1	Yes	Yes
3	Isointense	Hyperintense	Hyperintense	Hyperintense	Peripheral & Homogenous	1	No	Yes
4	Isointense	Hyperintense	N/A	N/A	N/A	3	Yes	Yes
5	Slightly Hyperintense	Hyperintense	N/A	N/A	Peripheral & Homogenous	1	Yes	No
6	Isointense	N/A	N/A	N/A	Peripheral & Homogenous	1	No	No
7	Slightly Hyperintense	Hyperintense	Hyperintense	Hyperintense	Peripheral & Homogenous	2	Yes	Yes
8	Isointense	Hyperintense	N/A	N/A	Homogeneous	1	No	Yes
9	Isointense	Hyperintense	Hyperintense	N/A	N/A	3	Yes	No
10	N/A	Hyperintense	N/A	N/A	N/A	4	No	Yes
11	Isointense	Hyperintense	N/A	N/A	N/A	2	No	Yes
12	Isointense	Hyperintense	Hyperintense	N/A	Inhomogeneous	3	No	Yes
13	Isointense	Hyperintense	Hyperintense	Hyperintense	Peripheral & Homogenous	2	Yes	Yes
14	Isointense	Hyperintense	Hyperintense	N/A	Inhomogeneous	2	Yes	Yes
15	Isointense	N/A	Hyperintense	N/A	Peripheral & Homogenous	3	Yes	Yes
16	N/A	Hyperintense	N/A	N/A	N/A	2	No	No
17*	Isointense	Hyperintense	N/A	Hyperintense	Homogenous	No Muscle Involvement	No	No
18*	Isointense	N/A	N/A	Hyperintense	Peripheral & Homogenous	No Muscle Involvement	Yes	Yes
19*	Slightly Hyperintense	Hyperintense	Hyperintense	N/A	Peripheral & Homogenous	1	No	Yes
20*	Isointense	Hyperintense	Hyperintense	N/A	Homogenous	No Muscle Involvement	No	No
21*	Isointense	Hyperintense	Hyperintense	N/A	Peripheral & Homogenous	1	No	No
22*	Isointense	Hyperintense	N/A	Hyperintense	Peripheral & Homogenous	No Muscle Involvement	No	No
23*	Isointense	Hyperintense	N/A	N/A	Inhomogeneous	1	No	Yes
24*	Isointense	Hyperintense	Hyperintense	N/A	N/A	No Muscle Involvement	No	No
25*	Isointense	N/A	Hyperintense	Hyperintense	Homogenous	1	No	No
26*	Isointense	Hyperintense	N/A	N/A	Inhomogeneous	No Muscle Involvement	No	Yes
Total	21 Isointense 3 Slightly Hyperintense 2 N/A^#^	21 Hyperintense 5 N/A	14 Hyperintense 12 N/A	7 Hyperintense 19 N/A	11 Peripheral & Homogenous 5 Homogeneous 4 Inhomogeneous 6 N/A	9 Single 11 Multiple 6 No Muscle Involvement	Yes 10 (38%) No 16	Yes 15 (58%) No 11

^1^ Compared to adjacent skeletal muscle

*subcutaneous manifestations

# not applicable

**Fig. 1:**
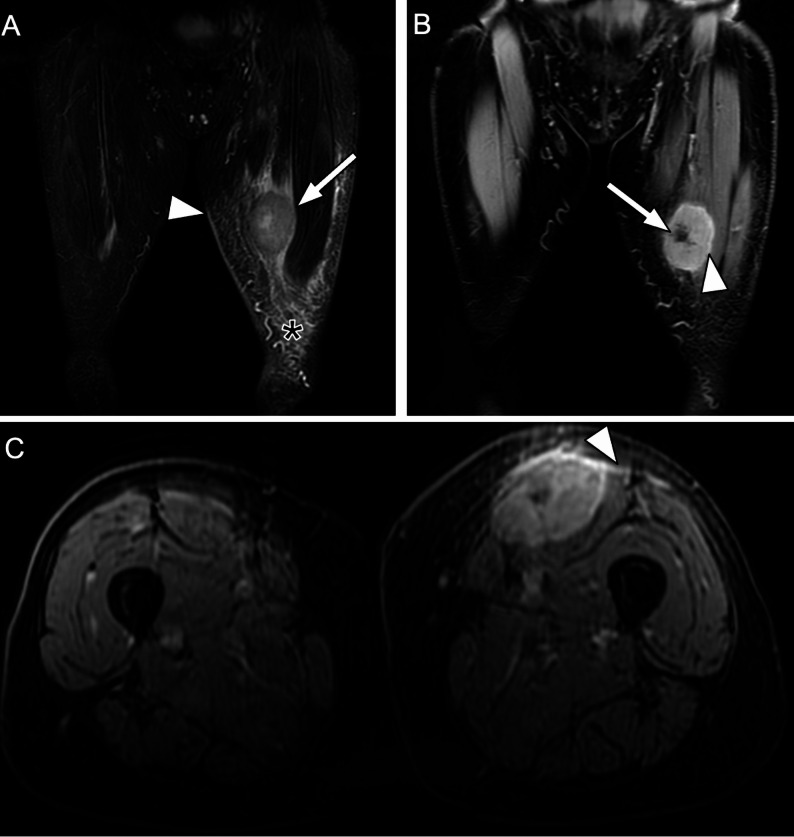
MRI of a 79-year-old female presenting with non-Hodgkin lymphoma manifestation in the left thigh. **A** Coronal STIR image shows a hyperintense tumor (arrow) with associated enlargement of the affected extremity. Furthermore, subcutaneous stranding (asterisks) and skin thickening (arrowhead) can be noted. **B** Coronal contrast enhanced fat-saturated T1-weighted image reveals central necrosis (arrow) and peripheral contrast enhancement (arrowhead). **C **Transversal contrast enhanced fat-saturated T1-weighted image reveals signal enhancement of adjacent fascia lata (arrowhead)

**Fig. 2:**
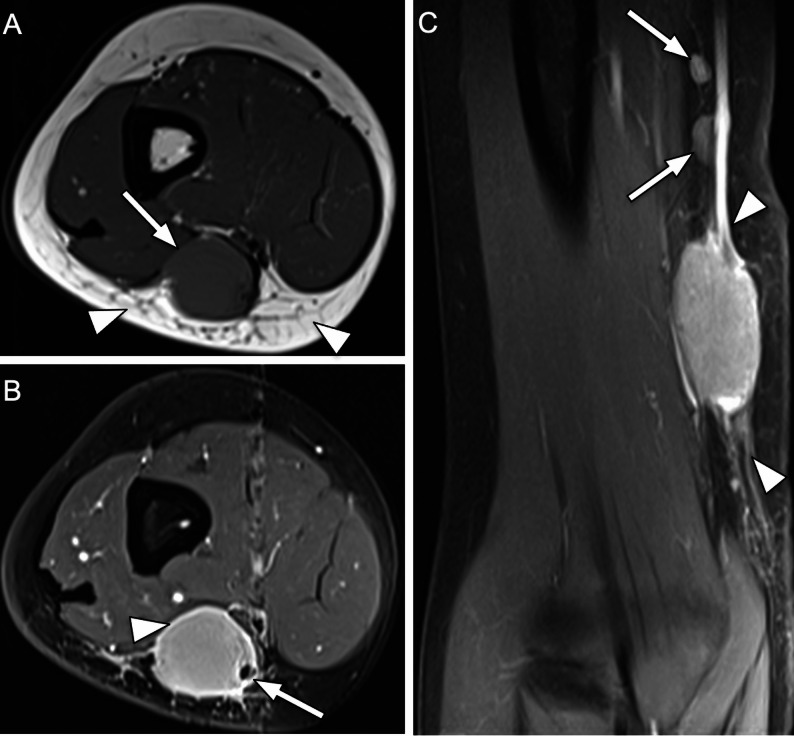
MRI of a 44-year-old female presenting with non-Hodgkin lymphoma manifestation in the upper arm. **A** Transversal T1-weighted image shows a subcutaneous tumor (arrow), presenting with slightly hyperintense signal intensity compared to muscle and subcutaneous stranding (arrowheads) **B** Transversal contrast enhanced fat-saturated T1-weighted image shows peripheral enhancement (arrowhead) accompanied by slightly less intensive homogenous enhancement of the remaining tumor. Additionally, encasement of the basilic vein (arrow) is recognizable. **C** Coronal contrast enhanced fat-saturated T1-weighted image shows growth oriented along the basilic vein (arrowheads) and close association of two additional lesions (arrows) with the basilic vein

**Fig. 3:**
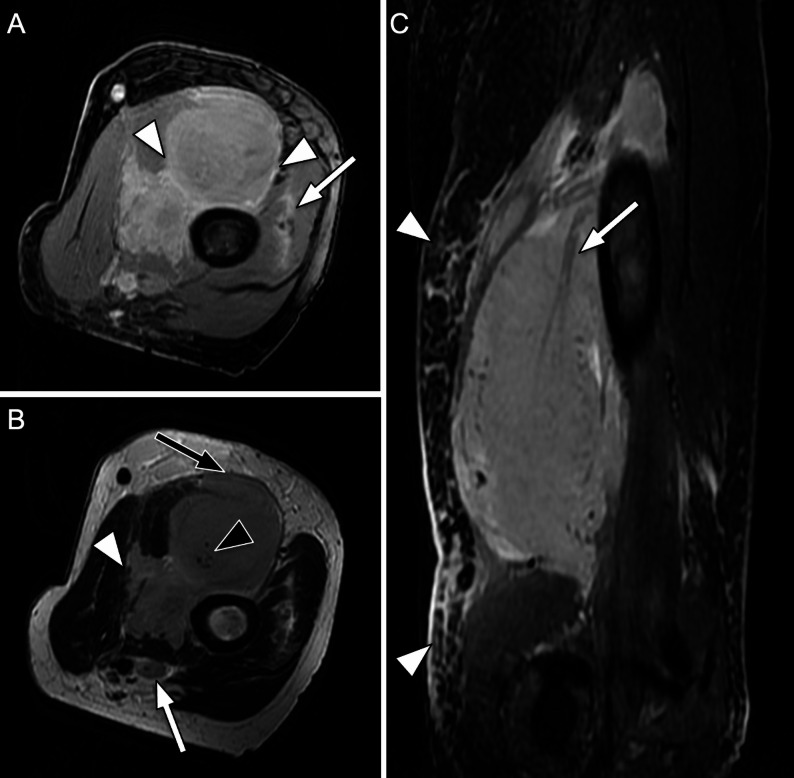
MRI of a 73-year-old male presenting with non-Hodgkin lymphoma manifestation in the upper arm. **A** Transversal contrast enhanced fat-saturated T1-weighted image shows partial peripheral enhancement (arrowheads) accompanied by slightly less intense homogenous enhancement of the remaining tumor. Moreover, one can note manifestation in the extensor compartment, encasing branches of the medial collateral artery (arrow). **B** Transversal T2-weighted image shows encasement of radial nerve and collateral radial artery (black arrowhead) and close association of an additional lesion to the brachial neurovascular bundle (white arrow). Moreover, co-existence of well-defined (black arrow) and ill-defined (white arrowhead) tumor margins can be recognized. **C** Coronal STIR image shows subcutaneous stranding (arrowheads), long segmental involvement and longitudinally traversing deep brachial artery (arrow)

**Fig. 4:**
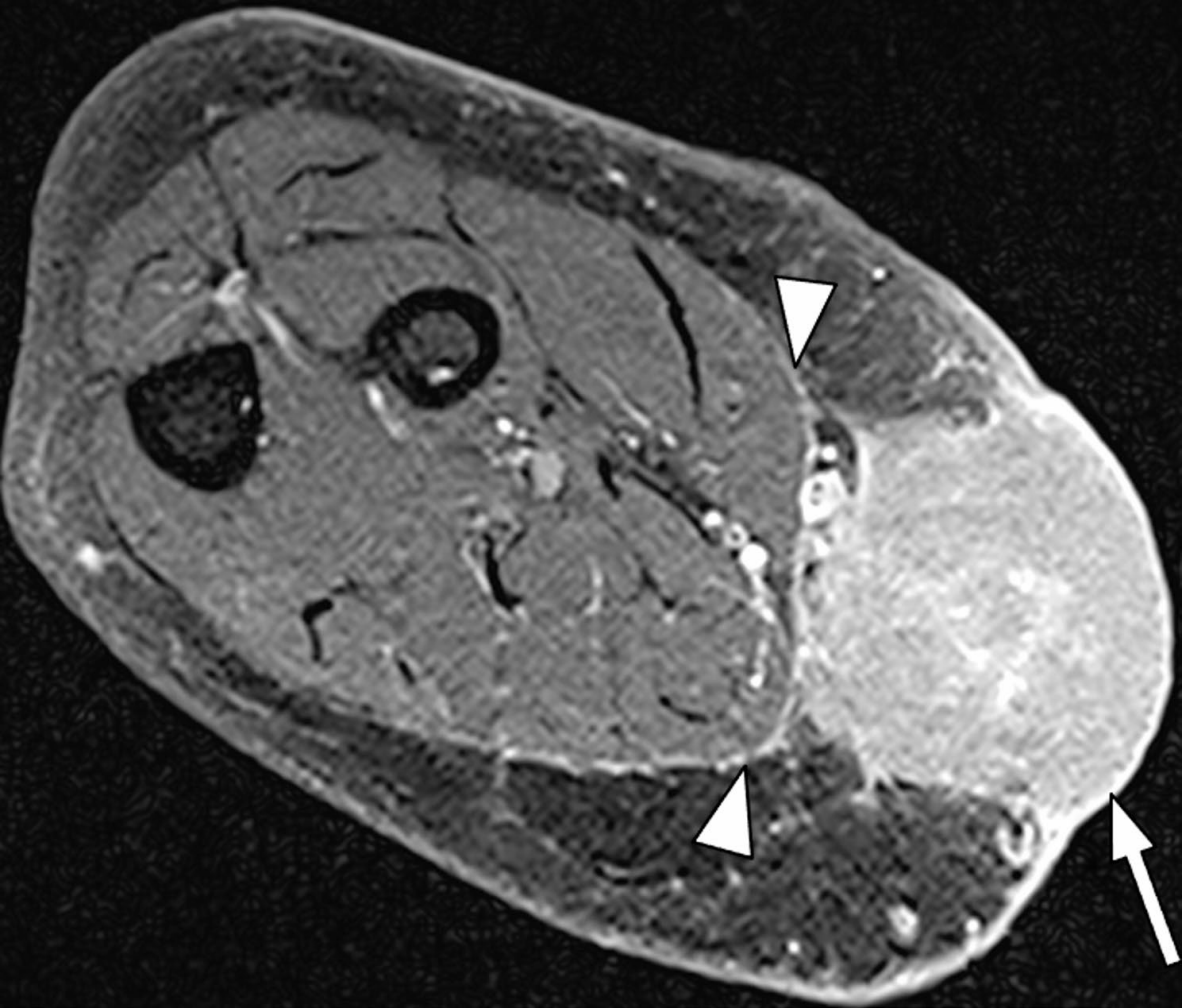
Transversal contrast enhanced fat-saturated T1-weighted image of a 55-year-old female shows a subcutaneous tumor (arrow) of hyper intense signal intensity and associated enhancement of adjacent fascia (arrowheads)

All T1w images (*n* = 24) showed iso- to slightly hyperintense signal intensity compared to adjacent skeletal muscle. In all water sensitive sequences (T2w (*n* = 21), PDw (*n* = 14), STIR (*n* = 7)) hyperintense signal intensity was identified. Fat suppressed T1w images without application of contrast agent were attained in five patients, of which four (80%) showed hyperintense signal intensity and one (20%) showed isointense signal intensity compared to adjacent muscle. Contrast enhanced images mainly showed predominant, partial or complete enhancement of peripheral tumor accompanied by slightly less intensive homogenous enhancement of the remaining tumor (*n* = 11/20; 55%) (Figs. 1, 2 and 3). Additionally, irregular enhancement of adjacent fascia was found in twelve cases (60%) (Figs. 1 and 4).

This finding was more frequent in PML manifestations (*n* = 8/11; 73%) than in PSCL manifestations (*n* = 4/9; 44%) (*p* = 0.36 ; CI=-12.9 to 59.3). Demarcation patterns of ASTL presentations showed co-existence of partially ill- and partially well-defined areas in 14 (54%) cases (Fig. 3). Solely ill-defined margins were found in nine (35%) cases and solely well-defined margins in three cases (12%).

**Fig. 5:**
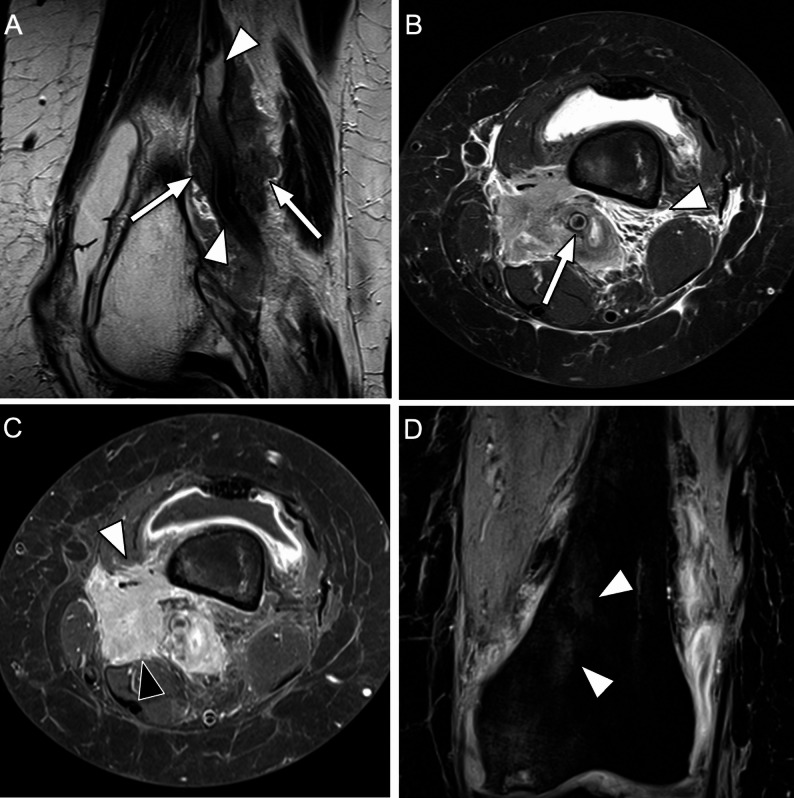
MRI of a 73-year-old female presenting with non-Hodgkin lymphoma manifestation in the popliteal fossa. **A** Sagittal T2-weighted image shows tumor (arrows) extending along the popliteal vessels (arrowheads). **B** Transversal fat-saturated PD-weighted image reveals encasement of popliteal vessels (arrow) and peritumoral edema (arrowhead). **C** Transversal contrast enhanced fat-saturated T1-weighted image shows infiltration of anterior (white arrowhead) and posterior (black arrowhead) femoral compartments **D** Coronal contrast enhanced T1-weighted image reveals patchy enhancement of medullary space (arrowheads) without cortical destruction

20 patients presented muscle involvement of ASTL, either as primary intramuscular manifestation (*n* = 16) or as secondary infiltration originating from primary subcutaneous manifestations (*n* = 4). Differences in numbers of affected muscles, affected compartments, tissue types and long segmental growth pattern showed statistically significant differences between PML and PSCL (Table 2). Multiple involved muscles were identified in all cases of PML, whereas this finding was present in one of four PSCL manifesting with muscle involvement (*p* = 0.003 ; CI = 26.1 to 95.4) (Table 2). Multiple compartments were involved in 11 cases (68%) with PML (Figs. 3 and 5), compared to none of the PSCL involving muscle (*p* = 0.026 ; CI = 14.0 to 85.8).

Multiple tissue types were involved in 18 cases (69%) ASTL and were found more often in PML (*n* = 14; 88%) than in PSCL manifestations (*n* = 4; 40%) (*p* = 0.025 ; CI = 10.4 to 72.4).

Moreover, long segmental involvement was present in none of the PSCL, whereas this feature was found in 11 PML (69%) (*p* = 0.026 ; CI = 14.0 to 85.8) (Fig. 3). The presence of subcutaneous stranding was discernable in 15 cases (58%) (Figs. 1, 2 and 3) and was seen more frequently in PML (*n* = 11; 69%) than in PSCL (*n* = 4; 40%) (*p* = 0.228 ; CI=-8.9 to 57.5). Growth along neurovascular bundles and encasement of major vessels (Figs. 2, 3 and 5) displayed statistically significant differences between subgroups (*p* = 0.036, CI = 8.1 to 68.5; *p* = 0.046, CI = 3.6 to 67.4) (Table 2).

**Fig. 6:**
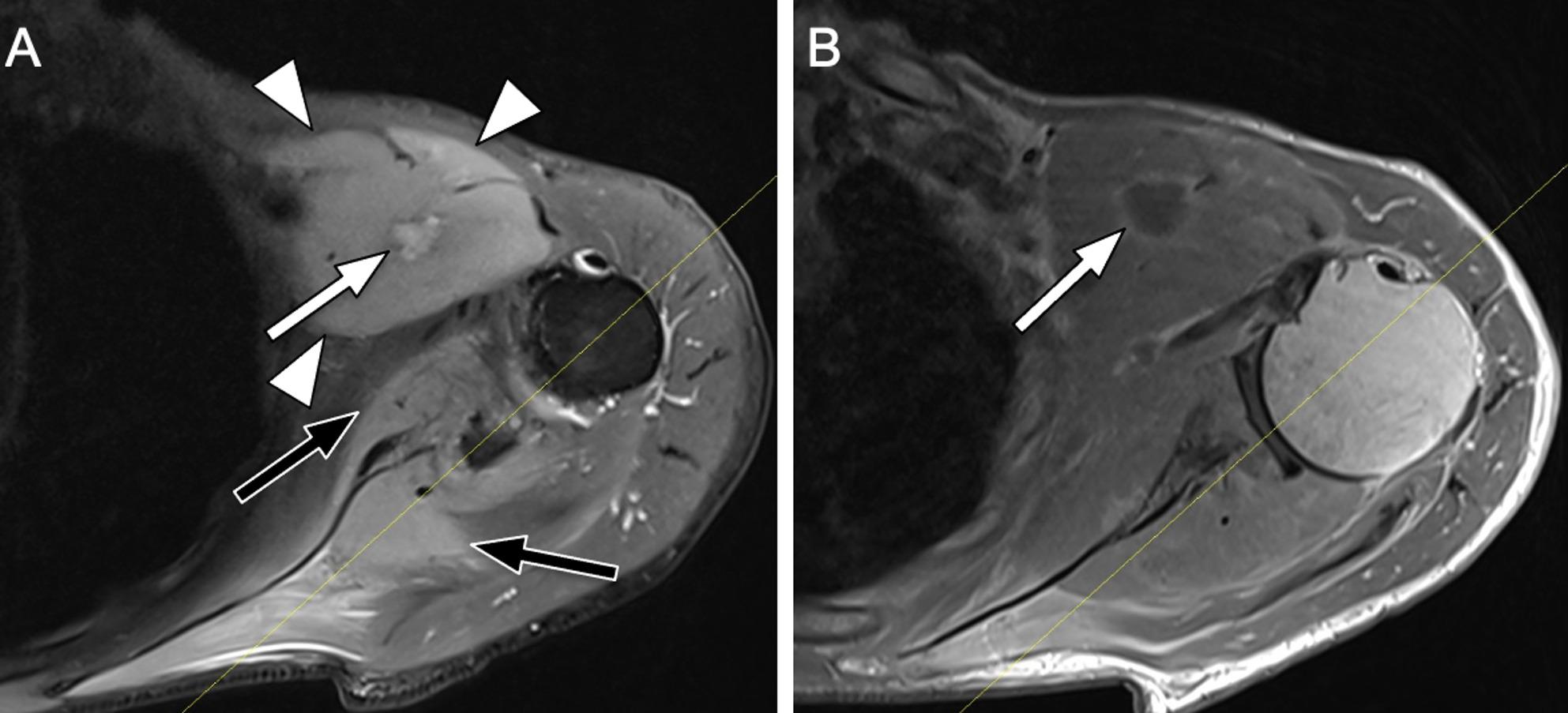
MRI of an 85-year-old female presenting with non-Hodgkin lymphoma of the shoulder. **A** Transversal PD-weighted image shows a hyperintense tumor (arrowheads), involving m. deltoideus, m. pectoralis major, m. infraspinatus and m. subscapularis (black arrows) and central signal alteration (white arrow). **B** Transversal contrast enhanced T1-weighted image reveals central necrosis (arrow)

In PML growth along neurovascular bundles was detected in nine cases (56%) and encasement of major vessels was recognizable in 13 cases (81%), whereas PSCL manifestations grew along neurovascular bundles in one case (10%) and showed encasement of major vessels in four cases (40%). Abnormal signal of bone marrow was observed in four cases (16%) of PML (Fig. 5), wherein one case showed cortical destruction and the remaining three cases showed uncompromised cortical bone. None of the PSCL showed signal alteration of bone. Traversing vessels were identified in 23 cases (88%). None of the investigated tumors showed encapsulation. Intratumoral necrosis (Figs. 1 and 6) was observed in four cases (15%) and was present solely in PML manifestations.

## Discussion

This study investigated MRI characteristics of ASTL in 26 patients, which represents the largest reported study investigating MRI characteristics of soft tissue lymphoma. MRI characteristics indicative of ASTL include hyperintense signal intensity in T2w-, PDw- and STIR sequences and iso- to slightly hyperintense signal intensity in T1w sequences compared to skeletal muscle. In contrast enhanced imaging predominant, partial or complete enhancement of peripheral tumor accompanied by slightly less intensive homogenous enhancement of the remaining tumor or isolated homogenous enhancement pose as indicators for the presence of ASTL. Moreover, irregular enhancement of adjacent fasciae may be indicative of ASTL. Additionally, regional lymphadenopathy, involvement of multiple muscles, compartments and tissues, long segmental involvement, growth along neurovascular bundles, encasement of major vessels, subcutaneous stranding, traversing vessels and the absence of encapsulation may be suggestive of ASTL in soft tissue tumor presentations.

Histologically, diffuse large B-cell lymphoma represented the most frequently identified subtype, which coincides with the literature and is in general the most prevalent subtype in lymphoma presentations [4, 26, 27]. Most frequently, the proximal extremities were involved (*n* = 22; 85%). Therein, a predilection for the lower extremities was discernable (*n* = 15; 58%), which is consistent with the literature [28]. A diameter larger than 5 cm was commonly measured (*n* = 20; 77%). This coincides with a T2 size in UICC TNM classification of appendicular soft tissue tumors [29]. Clinically our findings show that regional lymphadenopathy, local pain and in rare occasions B symptoms may be discernible. This coincides with existing literature [9, 30, 31]. Regional lymphadenopathy was identified in eight cases (31%). If present, this could be a first indicator for the presence of ASTL (Table 4) [6, 7, 9], as lymphadenopathy occurs in only 4% of STS [32] and metastatic spread to lymph nodes is reported in only 2–6% of appendicular STS [33–35].

**Table 4:** MRI features of ASTL compared with STS characteristics derived from literature

Feature	ASTL^#^	STS*
Lymphadenopathy/Nodal Metastasis	Common	Uncommon [32–35]
Contrast enhancement	Varying patterns	Heterogenous [46, 47]
Multicompartmental involvement	Common	Less frequent [4, 9, 1, 52]
Subcutaneous stranding	Common	Not described
Capsule	No capsule	Pseudocapsule may be present [53, 58–60]

* Data derived from literature

^#^ Data derived from this study

However, certain subentities such as epitheloid-, angio-, clear cell-, synovial- and rhabdomyosarcoma might show a higher predilection [36–38].

Elevation of LDH levels was a frequent finding in PML presentations (*n* = 9/11; 82%). This coincides with the literature [6, 31, 39, 40].

MRI revealed iso- or slightly hyperintense signal intensity in T1w sequences compared to adjacent skeletal muscle, whereas hyperintense signal intensity was found in all water sensitive sequences (T2, PD, STIR). These findings correspond with the current literature and seem to be a characteristic feature of ASTL [4, 5, 41–45]. Contrast enhanced images mainly showed predominant, partial or complete enhancement of peripheral tumor accompanied by slightly less intensive homogenous enhancement of remaining tumor (*n* = 11/20; 55%) or isolated homogenous enhancement (*n* = 5/20; 25%). A previous series presented by Chun et al. [26] reported peripheral thick bandlike enhancement in four (21%) and marginal septal enhancement in two of 19 investigated cases (11%). However, co-existence of predominant, partial or complete peripheral enhancement accompanied by slightly less intensive homogenous enhancement of the remaining tumor has not been reported before. Previous authors have reported both homogeneous and heterogeneous enhancement [4, 5, 16, 26].

However, if present, homogenous enhancement might pose an indicator of ASTL manifestation as STS is usually known to show heterogenous contrast enhancement [46, 47].

Irregular enhancement of adjacent fascial planes was a further frequently found feature of ASTL (*n* = 12; 60%). Consisting with our findings, a previous study reported thick irregular enhancement of fascial planes in 17 of 19 cases (89%) [26]. This finding might be associated with the infiltrative growth pattern of soft tissue lymphoma [26] and should be carefully differentiated from a tail sign possibly observable in certain subtypes of STS [48, 49].

Demarcation of ASTL presentations showed simultaneously existing partially ill- and partially well-defined areas in 14 cases (54%). This finding has been described in superficial soft tissue tumor presentations [50], however it has not been described in PML manifestations and might explain heterogenous reports in literature [16, 17].

Our findings with respect to the frequent identification of multiple involved muscles, multiple anatomical compartments, multiple involved tissue types and long segmental involvement were in line with the literature and pose as key indicators of ASTL [4, 5, 26, 30, 45]. Therein, the involvement of multiple compartments may be suggestive for the presence of ASTL rather than STS presentations, as soft tissue sarcoma is more likely to respect compartmental boundaries [4, 9, 51, 52].

Subcutaneous stranding poses as another common finding in ASTL, being present in 15 of our cases (58%) and being frequently reported by other authors [4, 13, 26]. This finding might be explained by reactive subcutaneous edema or lymphatic infiltration [4, 5, 42]. Furthermore, it might pose as a further asset to differentiate ASTL and STS as it has, to our best knowledge, not been described in STS presentations. However, it should be carefully differentiated from intralesional fat stranding (“dirty fat”), which might be present in atypical lipomatous tumors or liposarcoma [53, 54].

Growth along neurovascular bundles was present in nine cases (56%) and partial or complete encasement of major vessels in 13 cases (81%) of PML presentations. These findings are a previously reported aspect of soft tissue lymphoma [4, 5, 9, 55] and might be explained by tumor extension along lymphatic vessels, which accompany neurovascular bundles [4].

Four cases (16%) of PML showed signal alteration of bone marrow, wherein, only one (4%) showed cortical destruction. This finding is commonly known and might be explained by either edema or lymphomatous infiltration [4, 25, 30, 45, 56]. However, conflicting reports have been published as Chun et al. reported bone marrow involvement as absent in all 20 patients [26]. Cortical integrity seems to stay uncompromised in most cases, which might be due to spread through intracortical channels [57].

Traversing vessels were observed in 23 cases (92%) and pose as a further key feature of ASTL [26]. None of the investigated lesions showed signs of encapsulation. This represents a feature which has not been investigated by previous authors. However, pseudocapsule formation poses as a possible feature of STS [53, 58–60], hence the absence of a (pseudo-)capsule could serve as an indicator for the presence of ASTL.

Intralesional necrosis was observed in four cases (25%) presenting with PML. At the time of MRI attainment, biopsy or therapy had not been initiated in any of these patients. Our findings suggest that necrosis may occasionally occur in pretreatment ASTL, expanding the spectrum of reported MRI findings beyond current literature [25, 55, 56, 61], which describes general absence of necrosis and hemorrhage in MRI of patients presenting with pretherapeutic soft tissue lymphoma. To our best knowledge, intralesional necrosis was previously reported in merely three isolated cases [4, 7, 62] of intramuscular lymphoma investigated via MRI. Even though intralesional necrosis and hemorrhage is a well-known feature of STS presentations [11, 46, 53, 58, 63–67] we conclude that the presence of necrosis alone should not exclude soft tissue lymphoma from the differential diagnosis.

LDH levels, number of involved muscles, compartments and tissue types, the presence of long segmental involvement and involvement of major neuro-vascular structures were higher in PML and showed statistical significance between PML and PSCL (Table 2). These findings have not been observed before and might be explained by earlier recognition of superficial tumors and therefore reduced duration of pathological expansion. However, these subgroup findings should be interpreted with caution. The relatively small and unequal subgroup sizes resulted in limited statistical precision, as reflected by the wide confidence intervals of several effect estimates (Table 2). Thus, while the observed differences may indicate distinct imaging patterns of intramuscular and subcutaneous appendicular lymphoma manifestations, the exact clinical relevance of these differences remains uncertain.

In several soft tissue tumor entities it was shown that radiomics and machine-learning approaches allow the extraction of quantitative imaging features, which may surpass the information gathered by visual image interpretation. Therefore, these approaches could be of complementary use for differentiating benign from malignant lesions, estimating tumor grade and predicting treatment response [68–70]. Similar approaches could potentially be applied in ASTL diagnostics, partly by integrating the morphological indicators identified in this study. However, given the rarity of ASTL, heterogenous literature and the need for thorough external validation, such methods will need further investigation and larger sample sizes. 

As several conventional MRI features of STS have been shown to correlate with histological grade and patient outcome [46, 47, 67], a similar association may also exist in ASTL. Future studies should therefore investigate whether specific morphological MRI features of ASTL correlate with lymphoma subtype, treatment response or patient outcome.

### Practical imaging approach

**Fig. 7:**
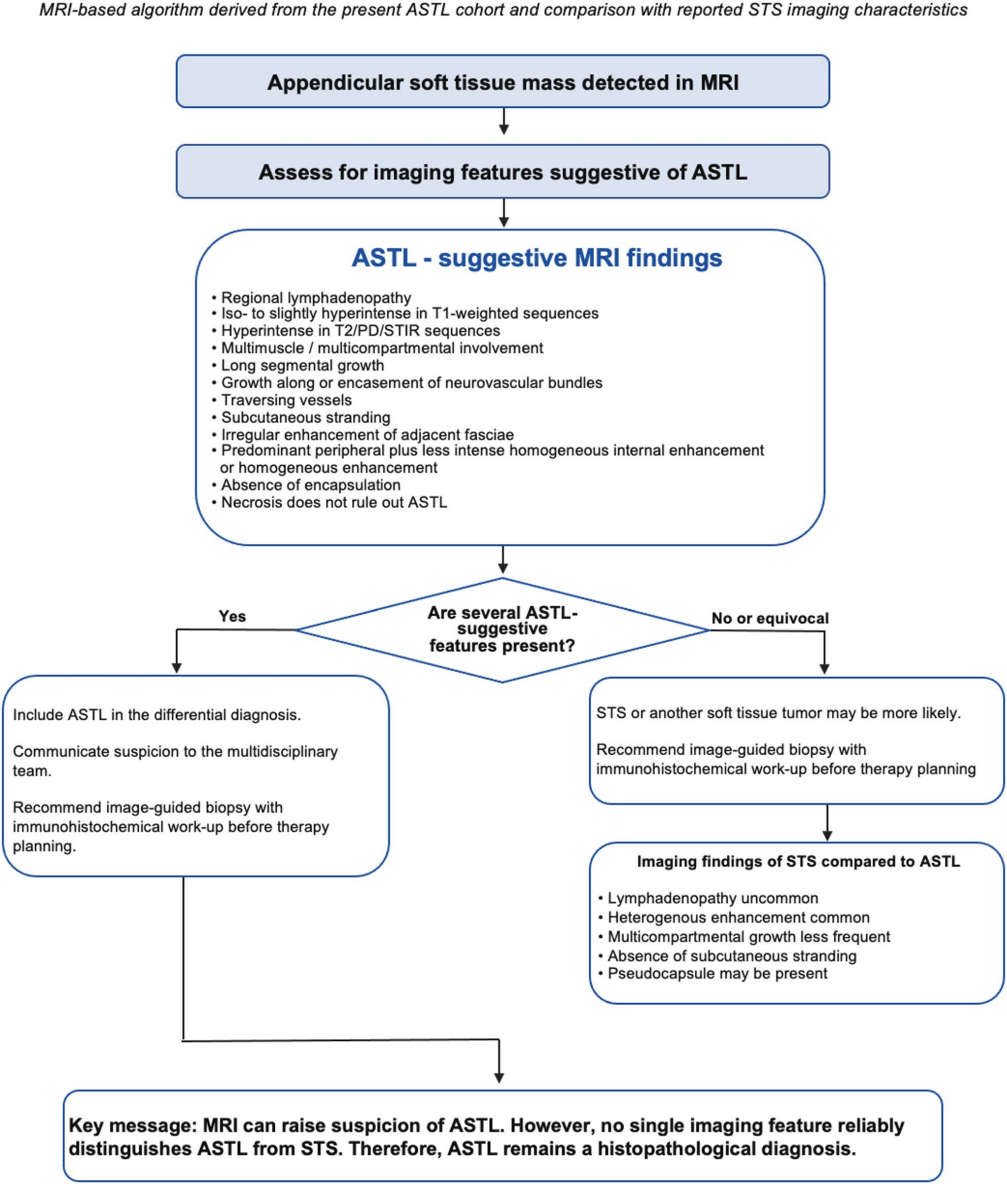
Practical imaging approach for suspected appendicular soft tissue lymphoma

Based on the findings of this study, we propose a practical MRI-based approach to raise suspicion of ASTL in patients presenting with appendicular soft tissue masses (Fig. 7). Radiologists should consider ASTL when several suggestive findings coexist, including lymphadenopathy, multimuscle or multicompartmental involvement, long segmental infiltrative growth, growth along or encasement of neurovascular structures, traversing vessels, subcutaneous stranding, iso- to slightly hyperintense signal intensity on T1-weighted images and hyperintense signal intensity on water-sensitive sequences, predominant, partial or complete peripheral enhancement accompanied by slightly less intense homogeneous enhancement of the remaining tumor after contrast administration, irregular fascial enhancement and absence of encapsulation. Importantly, no single imaging feature should be regarded as sufficient to reliably distinguish ASTL from STS. Instead, the overall constellation of available clinical and imaging features should be assessed to determine whether ASTL should be included in the differential diagnosis. MRI findings alone are not yet sufficient to establish a definitive diagnosis and image guided biopsy followed by an immuno-histological work-up should remain the diagnostic standard for definitive entity identification and treatment stipulation [71]. Therefore, the presented algorithm should be considered as a tool to support diagnostic awareness rather than a definitive diagnostic pathway.

## Limitations

Our study is limited by the retrospective study design, as knowledge of diagnosis prior to investigation could favor detection bias. The inclusion of varying MRI protocols is owed to the tertiary referral status of our institution and could be seen as a further limitation, nevertheless, a previous study observed no apparent differences in ASTL imaging between 1.5 and 3 Tesla systems [26]. 

Diffusion-weighted imaging (DWI) and apparent diffusion coefficient values (ADC) were not evaluated. Although DWI has been reported as useful in the assessment of skeletal muscle lymphoma [43, 72], it was available in solely one patient of the presented cohort. This is mainly attributed to the retrospective study design, the inclusion period beginning in 2011 and the heterogeneous MRI protocols available in a tertiary referral setting. Therefore, the absence of DWI/ADC assessment may have resulted in a loss of potentially relevant imaging information.

The absence of a control group investigating STS or other soft tissue tumors poses as a further limitation. Therefore, the imaging features presented in this study should not be interpreted as directly validated differentiation criteria between ASTL and STS. Future, prospective studies are needed to further validate if the presented MRI features can reliably discriminate ASTL from STS.

## Conclusion

Most often MRI of ASTL is misinterpreted as STS. However, MRI provides the above presented morphological indicators, that should raise suspicion of ASTL. Moreover, our findings suggest that necrosis may occasionally occur in pretreatment ASTL, and its presence alone should not exclude lymphoma from the differential diagnosis.

## Data Availability

The data attained in this study are available within the article.

## Abbreviations

ASTL: Appendicular soft tissue lymphoma
STS: Soft tissue sarcoma
PML: Primary intramuscular appendicular lymphoma
PSCL: Primary subcutaneous appendicular lymphoma
DWI: Diffusion-weighted imaging
ADC: Apparent diffusion coefficient
